# Somatostatin analogues labeled with copper radioisotopes: current status

**DOI:** 10.1007/s10967-017-5323-x

**Published:** 2017-06-13

**Authors:** Aleksandra Marciniak, Justyna Brasuń

**Affiliations:** Department of Inorganic Chemistry, Wroclaw Medical University, Borowska 211A, 50-556 Wroclaw, Poland

**Keywords:** Somatostatin, Somatostatin analogues, Copper radionuclides, Radiopharmaceuticals

## Abstract

Peptide receptor radionuclide therapy (PRRT) is a promising way to treat patients with inoperable tumors or metastatic neuroendocrine tumors. This therapeutic strategy is using radiolabeled peptides, which are capable of selective biding to receptors overexpressed in the cancer cells. One of the group of receptor-avid peptide used in the PRRT are the analogues of somatostatin (SST) connected to the complexes of radionuclides (e.g. ^90^Y, ^177^Lu or ^111^In). Many studies have shown that radiopharmaceuticals based on Cu radioisotopes are promising for the diagnosis and treatment of various cancers. This mini-review focuses on recent developments and summarises the results of multiple studies addressing SST agonists and antagonists radiolabeled to Cu radioisotopes.

## Introduction

Somatostatin (SST) is a naturally occurring peptide hormone first isolated in the 1970s from sheep hypothalamus [1]. In the human body, SST is found in the central nervous system, mainly in the hypothalamus, but may also be present in the dorsal root ganglia and sensory nerves, liver, lungs, pancreas, thyroid, small intestine, bone marrow, and adrenal glands [2–4]. In the central nervous system, SST acts as a neurotransmitter and neuromodulator. In the anterior pituitary gland, SST acts as a strong inhibitor of growth hormone and thyrotropin (TSH). It is also modulates cell proliferation, inhibits the secretion of insulin and glucagon in the gastrointestinal system, and affects peristalsis and the absorption of nutrients and ions. SST is active in the immune system, influencing the proliferation of lymphoid cells and the formation of immunoglobulins and cytokines [2, 4–6]. It is also noteworthy that SST has demonstrated an anti-proliferative activity on certain cancer cells [7].

**Fig. 1:**
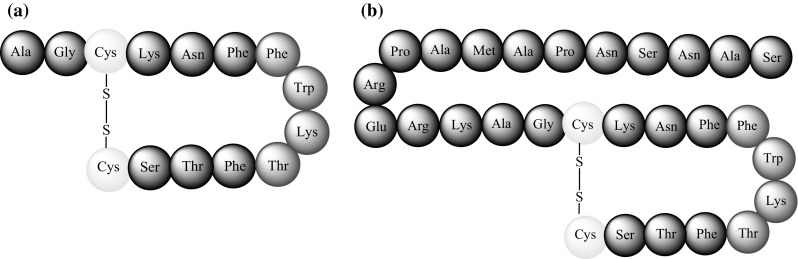
Schematic structure of **a** SST-14, **b** SST-28 [4]

SST exists in two bioactive forms, SST-14 and SST-28, characterised by the presence of a cyclic motif obtained by the formation of a disulphide bond between two cysteinyl moieties (Fig. 1a, b) [4].

It has been shown that four amino acids, Phe, Trp, Lys, and Thr (Fig. 1), located in the cyclic motif, are responsible for the biological activity of both naturally occurring forms of SST. Studies by Patel [2] have shown that while the presence of Trp and Lys is necessary for biological function, Phe and Thr can be replaced by other amino acids, e.g., Phe for Tyr and Ser to Thr or Val, without significantly impacting biological activity [2].

The biological activity of native hormones and their analogues is based on their interaction with somatostatin receptors (sstr). Five types of sstr have been defined (sstr1–sstr5). These are G-protein coupled receptors, encoded by different genes located on different chromosomes [2, 8–10]. The extracellular component of an sstr is responsible for its binding to the target peptide while the intracellular component is responsible for signal transduction [9]. Note that sstrs have been identified in both normal and pathological cells. The overexpression of sstr is commonly observed on the surface of cancer cells, e.g., pituitary tumours, neuroendocrine tumours, kidney cancer, colon cancer, glioblastoma, meningioma, and many others. However, the occurrence of different types of receptors is diverse [9].

**Fig. 2:**
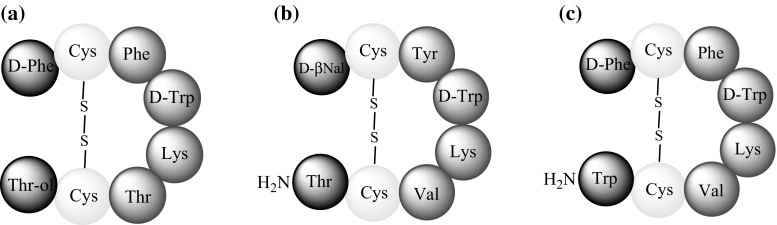
Schematic structure of: **a** octreotide, **b** lanreotide, **c** vapreotide [13]

Despite the wide spectrum of SST action, its application in vivo and in vitro is limited by its relatively short half-life of 1–3 min [11]. To address this limitation, effective SST analogues with longer half-lives were synthesised in the 1980s: ocreotide, lanreotide, and vapreotide (Fig. 2a, b, c). These compounds are still used in the diagnosis and treatment of a variety of illnesses [4, 11–13]. The characteristic feature of these compounds is the presence of a peptide chain fragment responsible for the biological activity of the native hormone. Shortening the peptide chain and inserting d-amino acids inhibits proteolysis and extends half-life. These alterations have significantly broadened the application range of SST analogues in medicine [14, 15].

**Fig. 3:**
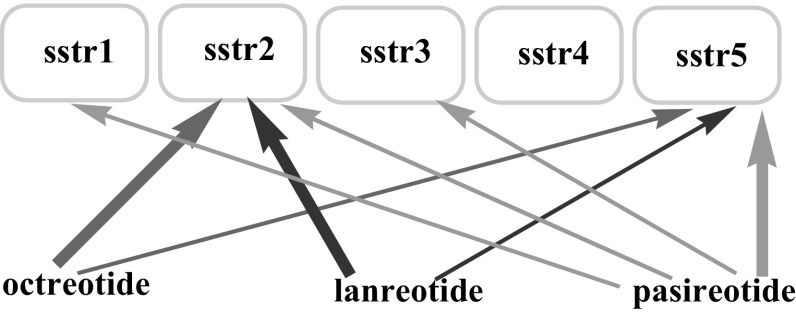
The affinity of octreotide, lanreotide and pasireotide to sstr [3]

The native hormone has a high affinity for all types of sstr [16], but these synthetic SST analogues have affinity for only specific receptors (Fig. 3) [3].

**Fig. 4:**
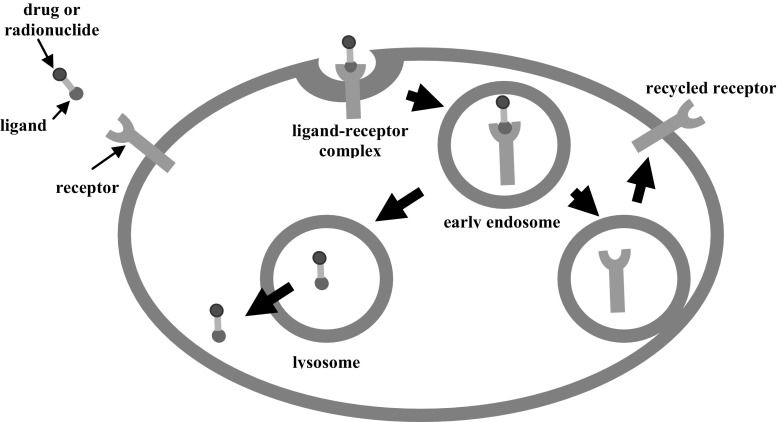
Scheme of the process of the internalization by receptor-mediated endocytosis [17, 18]

After binding of the hormone or its analogue to the receptor, the resulting complex is easily internalised by the cell (Fig. 4). This process, called receptor-mediated endocytosis, is the primary means of cellular penetration by these compounds [17, 18].

**Fig. 5:**
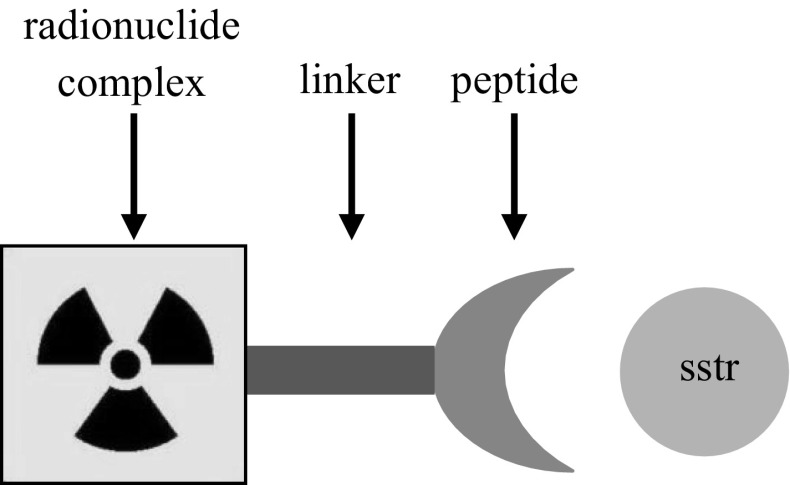
Construction of peptide-based radiopharmaceuticals [13]

When bound to radionuclides, synthetic agonists of sstr can be used in nuclear medicine in receptor-targeted diagnoses and therapies. Peptide-based radiopharmaceuticals are built from three components: a peptide, linker, and radioisotope complex (Fig. 5) [10].

The radioisotope complex is formed between a radionuclide, e.g., ^111^In or ^90^Y [10], and organic ligands such as DOTA, TETA, or DTPA, which act as chelators [12, 19]. It is important to note that it is not only the type or sequence of peptide that can affect the affinity of a SST analogue for sstr. In a radioisotope complex, modifications to the chelator, linker, or radioactive isotope can alter interactions with different types of receptors [19, 20].

OctreoScan (^111^In-DTPA-d-Phe^1^-octreotide) was the first radiopharmaceutical containing a SST analogue approved by the United States Food and Drug Administration in 1994 as an agent for the diagnosis of neuroendocrine tumours [21, 22]. However, clinical applications of OctreoScan were limited by its high affinity only to sstr2 and sstr5, making it unsuitable for imaging tumours expressing other receptors [20].

At higher doses, OctreoScan was also used in peptide receptor radionuclide therapies (PRRTs) [23]. However, somatostatin analogs radiolabeled with radioisotopes such as ^90^Y and ^177^Lu yielded better results than those radiolabeled with ^111^In [24–26]. Results obtained from the application of PRRT to patients with neuroendocrine tumours have been satisfactory and promising. Therefore, new SST analogues having high affinities for all five types of sstr are still being actively explored.

## Copper radionuclides in medicine

Natural copper occurs in two stable isotopes: more than 69% as ^63^Cu and about 31% as ^65^Cu [27]. There are 27 known radioisotopes of copper. Six of them are potentially useful in medicine, ^60^Cu, ^61^Cu, ^62^Cu, ^64^Cu for diagnosis, and ^64^Cu, ^66^Cu, ^67^Cu in targeted radiotherapy [27–29]. Their physical properties and means of application are presented in Table 1.

**Table 1:** Characteristics of medically important copper radioisotopes [27–29]

Radionuclide	Half-life time	Application	Radiation	Source
^60^Cu	23.7 min	Diagnosis	β^+^, γ	Cyclotron
^61^Cu	3.32 h	Diagnosis	β^+^, γ	Cyclotron
^62^Cu	9.7 min	Diagnosis	β^+^, γ	Generator/cyclotron
^64^Cu	12.7 h	Diagnosis/therapy	β^+^, β^−^, γ	Reactor/cyclotron
^66^Cu	5.4 min	Therapy	β^−^	Reactor/cyclotron
^67^Cu	61.4 h	Diagnosis/therapy	β^−^, γ	Reactor/cyclotron

Currently there are no radiopharmaceuticals containing copper radioisotopes that are acceptable for widespread use in humans [28], although some variants have yielded promising results in preclinical and clinical trials. The well-known coordination chemistry of copper simplifies the search for new radiopharmaceuticals incorporating Cu-radioisotopes in their structure. The resulting complexes can be potentially attached to antibodies, proteins, peptides, and other small, biologically important molecules [27, 29, 30].

Copper radionuclides exhibit several features that make them potentially useful in medicine.

^60^Cu and ^60^Cu are emitters β^+^-radiation (>93% [27, 28]) and are therefore potentially useful in positron emission tomography (PET). Moreover, due to the short half-life time they are especially useful if one considers the characteristically faster kinetics of smaller ligands [31]. However, due to relatively high positron energy and emission of γ radiation of ^60^Cu, copper radioisotope ^62^Cu is potentially more preferred as PET imaging agent [28]. ^61^Cu has a longer half-life than ^60^Cu and ^62^Cu, making it particularly appropriate for nuclear medicine and studies of copper metabolism. Furthermore, it can be used to image slow biological processes [32]. However, the plausibility of widespread use is limited by the high cost of production [32, 33]. ^62^Cu exhibits several atypical properties that are still being investigated extensively [27]. It is a β^+^-emitter with almost 100% purity of radiation with a simultaneously short half-life. This nuclide can be produced in a ^62^Zn/^62^Cu generator system [31]. A medical cyclotron with proton- or deuteron-induced reactions on Ni targets can be used to produce all three of the aforementioned radionuclides [31].

^66^Cu is a β-emitter that is applicable to radiotherapies for the treatment of tumours larger than 1 cm [34]. ^67^Cu has the longest half-life of the copper isotopes, making it potentially useful for both diagnosis and treatment, but is difficult to produce [32, 35].

^64^Cu is the most studied and well-described radionuclide of copper. It is a versatile isotope with potential applications in studies of copper metabolism, biodistribution tracking of potential drugs, and PET imaging [28]. Due to its half-life, emitted radiation, and stable complex formation with chelating molecules, it is the most promising of the Cu radionuclides for use in medicine [27]. ^64^Cu can be produced in a reactor [36]. However, the Washington University School of Medicine has developed a new, low-cost method of producing ^64^Cu using a biomedical cyclotron [37].

## Copper chemistry

In copper chemistry, two oxidation states are dominant, Cu(I) and Cu(II). Thus, the coordination chemistry of copper is relatively simple and well-known [34]. Cu(I), with a *d* ^*10*^ configuration, forms stable complexes with soft donors in aqueous solution. These complexes usually adopt a tetrahedral geometry when the coordination number is four. With two or three donors, a respective linear or trigonal planar geometry is typical [34, 38]. Cu(II) with a *d* ^*9*^ configuration forms complexes with four, five, or six coordination points. Complexes with four donors usually exhibit a square-planar or tetrahedral geometry. In 5-coordinated and 6-coordinated complexes, square-pyramidal and octahedral geometries, respectively, are most common. Jahn–Teller distortion is often observed in tetrahedral and octahedral complexes [34, 38].

Cu(I) complexes are more labile toward ligand exchange than are Cu(II) complexes. Therefore, Cu(I) complexes usually do not exhibit satisfactory kinetic stability for use in radiopharmaceuticals. In contrast, Cu(II) forms thermodynamically stable and kinetically inert complexes with macrocyclic chelators. Therefore, the design of ligands for copper radiopharmaceuticals has focused on Cu(II) complexes [34, 38]. An overview of chelators that have been evaluated for this purpose has been provided by Smith [29].

## Somatostatin agonists with copper radionuclides

**Fig. 6:**
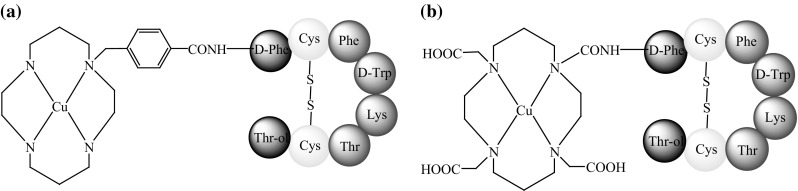
The structure of: **a** ^64^Cu-CPTA-d-Phe^1^-octreotide [39], **b** ^64^Cu-TETA-d-Phe^1^-octreotide [40]

SST agonists conjugated to copper radionuclide complexes behave similarly to the native hormone and the radioisotope is readily internalised by the cell [2]. As mentioned above, ^64^Cu is the most comprehensively studied copper radioisotope and complexes of ^64^Cu have been bound to SST analogues. Studies of two sstr agonists with ^64^Cu, TETA-d-Phe^1^-octreotide, and CPTA-d-Phe^1^-octreotide (Fig. 6) were published in 1995 [39] and described new conjugates for PET imaging. In vitro studies have been conducted using mouse brain cancer cells, and animal biodistribution studies have been carried out in rats with sstr-positive pancreatic tumours. The activities of these two SST analogues were compared with that of OctreoScan. The results showed that both TETA-d-Phe^1^-octreotide and CPTA-d-Phe^1^-octreotide had a greater affinity for sstr and a higher uptake in target tissue than did ^111^In-DTPA-d-Phe^1^-octreotide. The presence of macrocyclic, bifunctional chelating agents bound to the ^64^Cu-labeled analogues was the probable cause of the performance enhancement.

Also note that ^64^Cu-CPTA-d-Phe^1^-octreotide had a greater receptor binding affinity than TETA-d-Phe^1^-octreotide, perhaps due to the smaller size and greater lipophilicity of CPTA over TETA. However, ^64^Cu-TETA-d-Phe^1^-octreotide was characterised by a lower nephrotoxicity than ^64^Cu-CPTA-d-Phe^1^-octreotide, promoting further study [39].

Given the results described above, the effectiveness of ^64^Cu-TETA-d-Phe^1^-octreotide was evaluated in radiotherapy directed at rat pancreatic cancer cells [41]. The results showed that ^64^Cu-TETA-d-Phe^1^-octreotide was efficiently absorbed by the tumour cells with low toxicity and reasonable absorption in healthy organs. Therefore, there is a possibility of its potential use in targeted radiotherapies. Moreover, research has shown that dose fractionation is a more effective means of drug administration and that the application of two doses significantly enhanced the inhibition of tumour growth [41].

^64^Cu-TETA-d-Phe^1^-octreotide was also evaluated for its use in the diagnosis of neuroendocrine tumours by PET imaging [42]. Eight patients participated in the study: five with carcinoid tumours and three with islet cell tumours. PET imaging was performed using the copper radionuclide bound to new SST analogues or OctreoScan (as a reference). For two of the patients, better images were acquired with the ^64^Cu-TETA-d-Phe^1^-octreotide. In another study carried out on patients with neuroendocrine cancers, PET imaging with a ^64^Cu-SST analogue yielded better results than visualisation by scintigraphy with OctreoScan [43]. Unfortunately, it was also observed that the blood clearance of ^64^Cu-TETA-d-Phe^1^-octreotide was weak while the tumour clearance was rapid [44].

It was asserted above that small modifications to the peptide chain of a SST analogue can influence SST receptor affinity. The insertion of tyrosine instead of phenylalanine in the third position, or modification of the C-terminus, increased the degree of interaction between the SST analogue and the sstr [45–47]. This was shown in experiments performed with two SST analogues attached to ^64^Cu, TETA-Tyr^3^-octreotate (Fig. 6a), and TETA-octreotide (Fig. 5b) in rat and mouse animal models with SST receptor-positive pancreatic tumours [48]. Furthermore, primate imaging studies were performed by PET on a male baboon. In vivo and in vitro results showed a high affinity between sstr and ^64^Cu-TETA-Tyr^3^-octreotate. The uptake of radionuclide by the cancer cells was twice that of ^64^Cu-TETA-d-Phe^1^-octreotide, while elimination from the bloodstream was similar for both analogues. Therefore, ^64^Cu-TETA-Tyr^3^-octreotate is a promising radiopharmaceutical for the diagnosis of cancer with SST receptors. The increased uptake in target tissues would result in a lower dose administered to the patient, thereby reducing drug toxicity [48].

^64^Cu-TETA-Tyr^3^-octreotate was also found to induce tumour regression when used as a radiotherapeutic agent in baboon and male Lewis rats with pancreatic tumours [49]. Moreover, no lethal toxicity was observed even after multiple doses. However, significant differences in biodistribution were observed between rat and baboon models. Therefore, ^64^Cu-TETA-Tyr^3^-octreotate may not be suitable for use in targeted radiotherapies in humans [49].

**Fig. 7:**
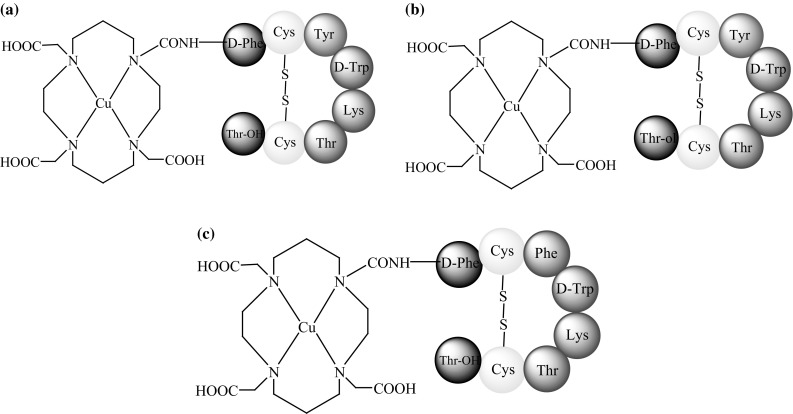
The structure of: **a** ^64^Cu-TETA-**Tyr** ^**3**^-octreotate, **b** ^64^Cu-TETA-**Tyr** ^**3**^-octreotide, **c** ^64^Cu-TETA-octreotate

Anderson and co-workers [44] published a study comparing four SST analogues conjugated to ^64^Cu complexes, TETA-octreotide (Fig. 6b), TETA-**Tyr** ^**3**^-octreotide, TETA-**Tyr** ^**3**^-octreotate, and TETA-octreotate (Fig. 7a–c). The study determined which modification, amino acid substitution at the third position (phenyloanaline by tyrosine) or a change at the C-terminus (substitution of Thr-ol by Thr-OH), has a greater influence on the binding affinity of ^64^Cu-TETA-**Tyr** ^**3**^-octreotate for sstr. In vitro studies showed that all of the analogues bound receptors with high affinity. However, the highest binding efficiency was observed with ^64^Cu-TETA-octreotate and ^64^Cu-TETA-**Tyr** ^**3**^-octreotate, while ^64^Cu-TETA-d-Phe^1^-octreotide exhibited the lowest binding efficiency. Therefore, C-terminus modification seemed to be more important for determining binding affinity than substitution at the third position.

Rat biodistribution studies showed the highest uptake of ^64^Cu-TETA-**Tyr** ^**3**^-octreotate and ^64^Cu-TETA-**Tyr** ^**3**^-octreotide by the adrenals, which supports the hypothesis that tyrosine is responsible for biological activity. The highest uptake of ^64^Cu-TETA-**Tyr** ^**3**^-octreotate was observed in the pituitary gland and pancreas. ^64^Cu-TETA-octreotate and ^64^Cu-TETA-**Tyr** ^**3**^-octreotide showed similar activities, while ^64^Cu-TETA-d-Phe^1^-octreotide showed the lowest degree of activity. Therefore, in this case, C-terminus modification and substitution at the third position had similar influences. Based on these conflicting results, it is difficult to clearly identify which of these modifications are more influential on binding affinity and bioactivity. Nevertheless, the best results were generally observed with ^64^Cu-TETA-**Tyr** ^**3**^-octreotate, making it the most promising agent for targeted radiotherapies [44].

**Fig. 8:**
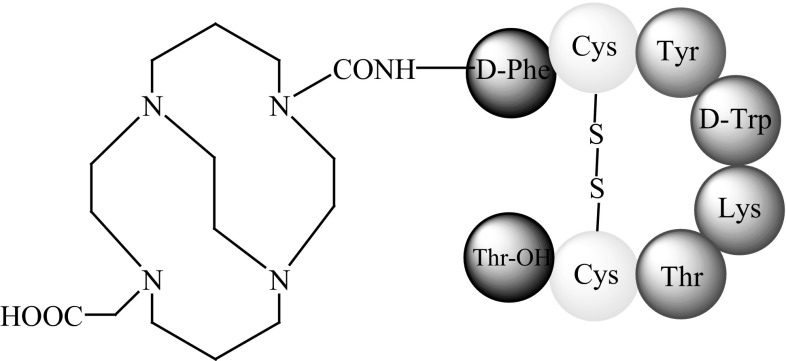
Structure of CB-TE2A-Tyr^3^-octreotate [50]

A new chelating agent, CBTE-2A (Fig. 8), has also been evaluated in conjunction with Tyr^3^-octreotate for its possible use in PET imaging [50]. The results were compared with those of ^64^Cu-TETA-Tyr^3^-octreotate (Fig. 7a).

Blood and liver clearance data, acquired in a tumour-bearing rat model, showed that CBTE-2A is a better complexing agent for the copper radionuclide than was TETA. In addition, ^64^Cu-CB-TE2A-Tyr^3^-octreotate showed greater accumulation in pathological cells, thereby increasing the sensitivity of tumour detection by PET [50].

^64^Cu-CB-TE2A-Tyr^3^-octreotate is also a promising agent for targeted radiotherapy [51]. Despite having a similar binding affinity for sstr as ^64^Cu-CB-TE2A-Tyr^3^-octreotate and ^64^Cu-DOTA-Tyr^3^-octreotate, ^64^Cu-CB-TE2A-Tyr^3^-octreotate is more effectively internalised in a human colorectal cell line. This is likely due to the fact that DOTA is a weaker chelating agent than TE2A, which allows ^64^Cu to more easily dissociate before internalisation [51].

**Fig. 9:**
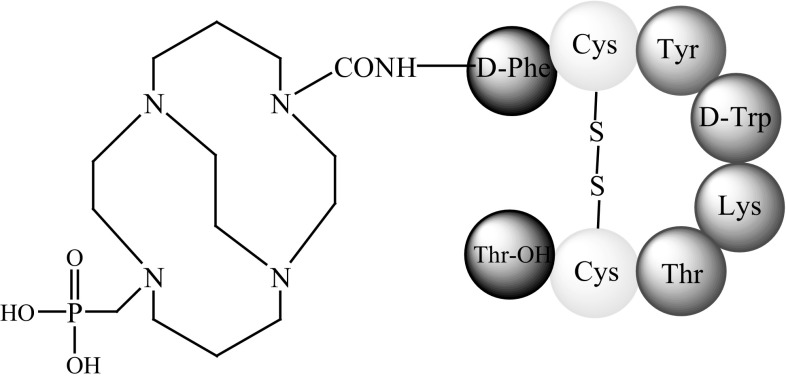
Structure of CB-TE1A1P**-**Tyr^3^-octreotate [53]

It has furthermore been shown that insertion of a phosphonate group into the chelating motif increases the thermodynamic stability [52] of the conjugated complex. Therefore, a new chelating agent, CB-TE1A1P, was synthesised and conjugated to Tyr^3^-octreotate (Fig. 9) for in vitro and in vivo studies [53].

Of these two new chelating agents, CB-TE1A1P was easier to radiolabel because it requires heating to only 40 °C for 60 min, while the radiolabelling of CBTE-2A is performed at 95 °C. Both analogues showed similar binding affinities for sstr2 in a tumour-bearing rat model, although ^64^Cu-CB-TE1A1P-Tyr^3^-octreotate exhibited a more suitable biodistribution for tumour detection by PET. Therefore, CB-TE1A1P is a promising chelator for use in copper-based radiopharmaceuticals [53].

**Fig. 10:**
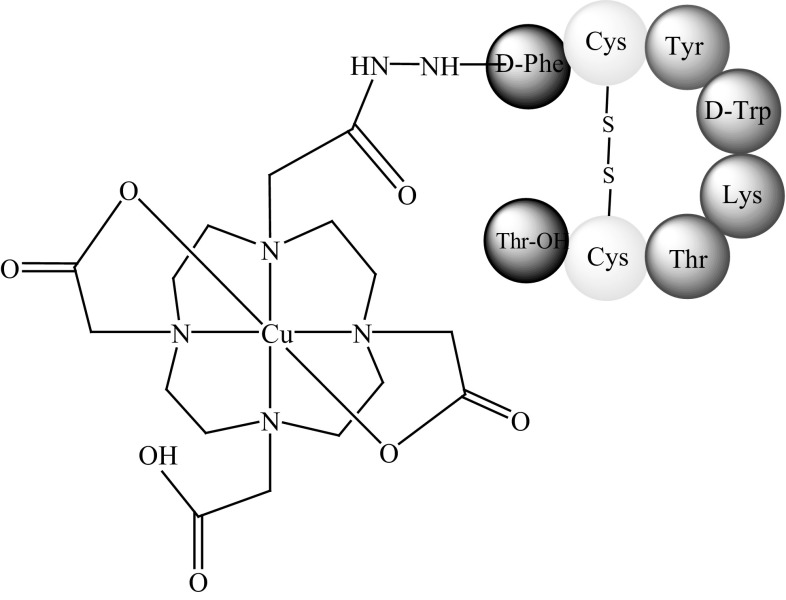
Structure of ^64^Cu-DOTA-Tyr^3^-octreotate

Pfeifer and co-workers [54–56] evaluated the possibility of using ^64^Cu-DOTA-Tyr^3^-octreotate (Fig. 10) for PET detection of neuroendocrine tumours. The results were then compared with the results of single-photon emission computed tomography (SPECT) using OctreoScan.

Imaging ^64^Cu-DOTA-Tyr^3^-octreotate yielded images with greater quality and a higher tumour-to-background ratio than OctreoScan. In the first study, a lower radiation burden was observed and additional lesions were identified in six of fourteen patients [54]. The second study showed that in 35 of 100 cases, PET utilizing a copper radiopharmaceutical allowed the visualisation of foci in organs not seen in SPECT utilizing gold standard [55]. In the third cited article, the authors describe a study of 112 patients with NETs [56]; once again, ^64^Cu-Tyr^3^-octreotate yielded better results than OctreoScan. In 84 of the patients, more lesions were observed in PET images acquired with the copper radiopharmaceutical. The accuracy and diagnostic sensitivity were also greater with ^64^Cu-Tyr^3^-octreotate than with ^111^In-DTPA-octreotide. In summary, the authors concluded that ^64^Cu-Tyr^3^-octreotate could be a suitable replacement for OctreoScan [55].

^64^Cu-Tyr^3^-octreotate was also compared with ^68^Ga-DOTA-Tyr^3^-octreotide in studies conducted with 59 patients presenting neuroendocrine tumours [57]. ^64^Cu-DOTA-Tyr^3^-octreotate showed 42 lesions not found in patients diagnosed with ^68^Ga-DOTA-Tyr^3^-octreotide, but diagnosis with ^68^Ga revealed 26 lesions not observed in PET images obtained with the copper SST analogue. The detection sensitivities for both analogues were identical. However, the authors concluded that ^64^Cu-Tyr^3^-octreotate was more favourable and easier to use in a clinical setting [57].

**Fig. 11:**
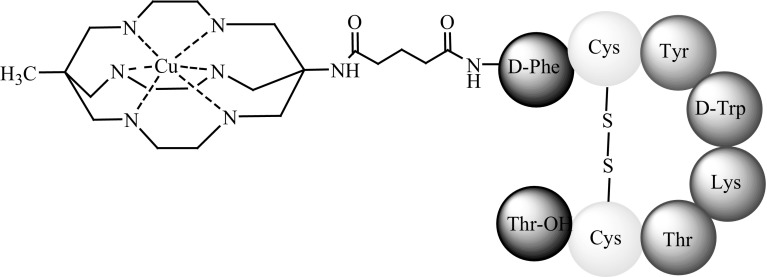
Structure of ^64^Cu-SAR-Tyr^3^-octreotate [58]

Peterson and coworkers [58] reported the design and synthesis of a new bifunctional ligand, MeCoSAR, and its conjugation to Tyr^3^-octreotate. The resulting SST analogue was radiolabelled with ^64^Cu (Fig. 11), and its in vitro and in vivo properties in A427-7 tumour-bearing Balb/c mice were compared against those of ^64^Cu-DOTA-Tyr^3^-octreotate. ^64^Cu-SAR-Tyr^3^-octreotate demonstrated a high selectivity for tumour cells presenting sstr2. Both radiopharmaceuticals showed good biodistribution and high-quality PET imaging. However, ^64^Cu-SAR-Tyr^3^-octreotate accumulated less in non-target organs than did ^64^Cu-DOTA-Tyr^3^-octreotate, making the former a more promising agent for imaging and therapy [58].

^64^Cu-DOTA-Tyr^3^-octreotate was also evaluated in a mouse pheochromocytoma model to determine cellular uptake, tumour binding, and functional in vivo imaging [59].

**Fig. 12:**
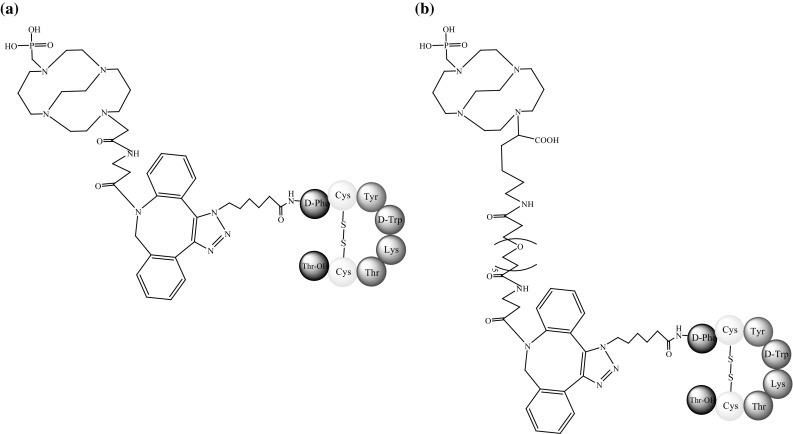
Structures of: **a** CB-TE1A1P-DBCO-Tyr^3^-octreotate, **b** CB-TE1K1P-PEG_4_-DBCO-Tyr^3^-octreotate [60]

Two new SST analogues, ^64^Cu-CB-TE1A1P-DBCO-Tyr^3^-octreotate and ^64^Cu-CB-TE1K1P-PEG_4_-DBCO-Tyr^3^-octreotate (Fig. 12), were examined following conjugation with copper chelators by strain-promoted click chemistry [60]. *In vitro* and in vivo studies were performed in tumour-bearing female mice with colon cancer and the results compared with those of ^64^Cu-CB-TE1A1P-Tyr^3^-octreotate. Both complexes exhibited a high degree of tumour-to-background contrast and tumour-specific uptake in PET/CT imaging, although better results were obtained with ^64^Cu-CB-TE1A1P-Tyr^3^-octreotate [60].

**Fig. 13:**
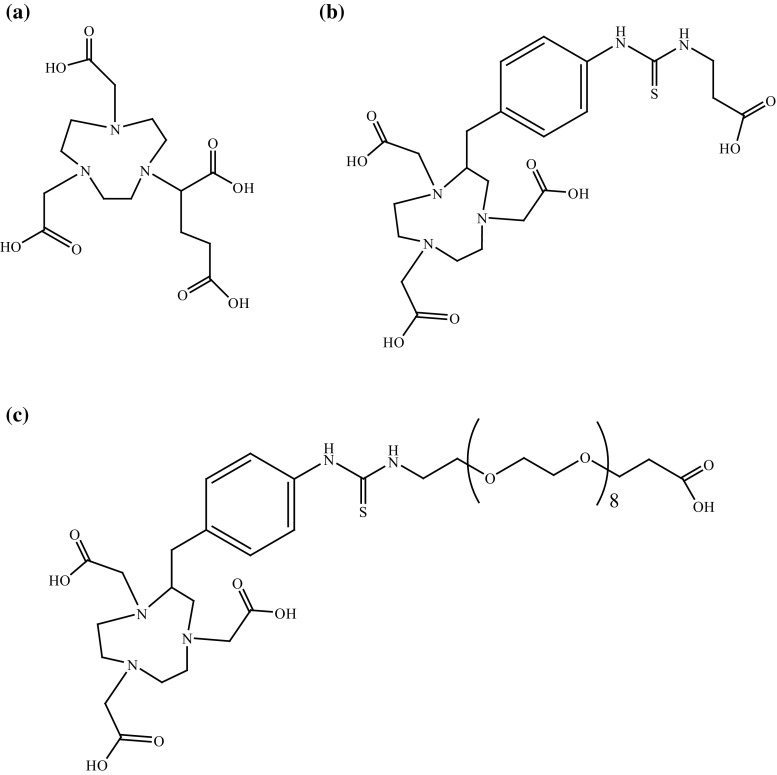
Structures of: **a** NODAGA, **b** NOTA-β-Ala, **c** NOTA-PEG_8_ [61]

Tyr^3^-octreotate was also conjugated to two commercially available chelating agents, p-SCN-Bn-NOTA and NODAGA, and radiolabelled with ^64^Cu [61] (Fig. 13). NODAGA was connected directly to Tyr^3^-octreotate, while p-SCN-Bn-NOTA was conjugated via β-Ala and PEG_8_ linkages (Fig. 13). The results obtained from in vitro and in vivo studies were compared with those of ^64^Cu-CBTE2A-Tyr^3^-octreotate and indicated that ^64^Cu-NODAGA-Tyr^3^-octreotate had the higher binding affinity. The length of the linker was demonstrably important, as ^64^Cu-NOTA-PEG_8_-Tyr^3^-octreotate had the lowest degree of uptake and internalisation in cells with sstr2. Both of these variables were greater with the smaller β-Ala linker or with no linker. Tumour cells showed similar uptake levels of ^64^Cu-NODAGA-Tyr^3^-octreotate, ^64^Cu-NOTA- β-Ala -Tyr^3^-octreotate, and ^64^Cu-CBTE2A-Tyr^3^-octreotate. In general, in vivo studies yielded comparable results for ^64^Cu-NODAGA-Tyr^3^-octreotate and ^64^Cu-CBTE2A-Tyr^3^-octreotate. The least favourable results were obtained with ^64^Cu-NOTA-PEG_8_-Tyr^3^-octreotate. Therefore, direct conjugation of the chelating agent to the SST analogue is the best way of designing new radiopharmaceuticals.

## Somatostatin antagonists with copper radionuclides

SST antagonists conjugated with radioisotopes became a promising tool in nuclear medicine after Mihaela Ginj and co-workers [56] showed that they are better than agonists for the in vivo targeting of tumours with sstr2 and sstr3 in tumour-bearing mouse models. This was a surprising result because antagonists do not internalise in tumour cells and antagonise the normal effects of receptor coupling to adenylyl cyclase [2, 62]. Initially, studies were performed with SST antagonists complexed with ^111^In, but radiocopper analogues were evaluated after the promising results described in the previous chapter. The first clinical application of SST antagonists was the use of ^111^In analogues in five patients with metastatic thyroid cancer in 2011 [63]. Since then, SST antagonists have become attractive agents for the diagnosis and treatment of cancer.

**Fig. 14:**
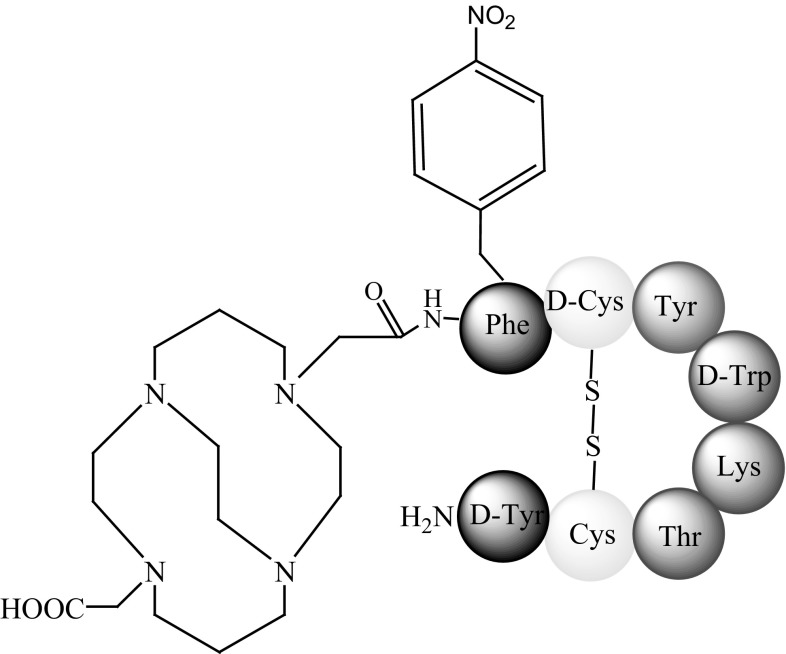
Structure of CB-TE2A-sstr-ANT (phenyloalanine moiety is modified) [64]

In 2008, the biological activity of ^64^Cu-CB-TE2A-sstr2-ANT, where ANT is Phe-4-NO_2_-c(d-Cys-Tyr-d-Trp-Lys-Thr-Cys)-d-Tyr-NH_2_ (Fig. 14), was assessed in in vitro and in vivo studies in a tumour-bearing rat model [64].

The activity of ^64^Cu-CB-TE2A-sstr2-ANT, an antagonist, was compared against that of ^64^Cu-CB-TE2A-Tyr^3^-octreotate, an agonist. The antagonist showed less internalisation than the agonist. ^64^Cu-CB-TE2A-sstr2-ANT boasted rapid blood clearance, but was slower than the agonist to clear the liver and kidneys. Tumour-to-blood and tumour-to-muscle ratios were both greater for ^64^Cu-CB-TE2A-sstr2-ANT and excellent PET images were obtained 4 h after injection. Thus, ^64^Cu-CB-TE2A-sstr2-ANT is an attractive and promising agent for use in nuclear medicine [64].

**Fig. 15:**
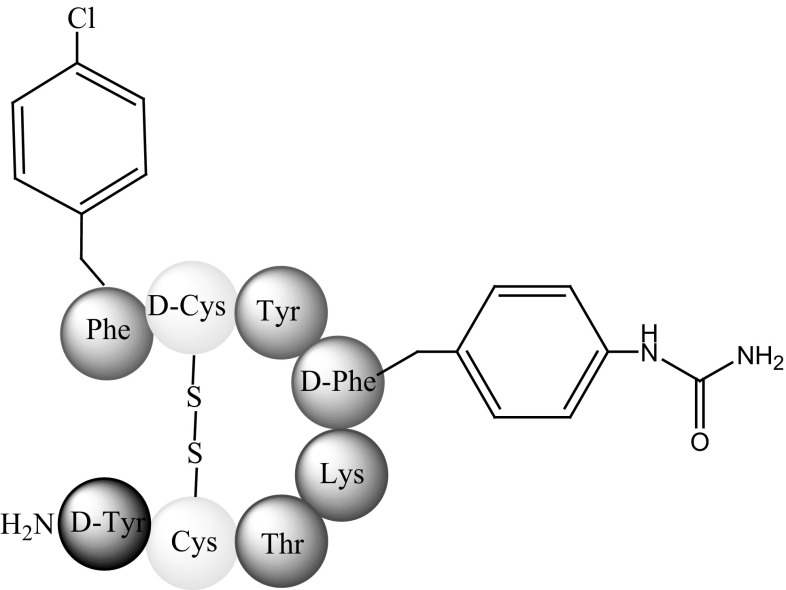
Structure of LM3 (phenyloalanine moieties are modified) [65]

Fani and co-workers [65] analysed a new SST antagonist LM3 (p-Cl-Phe-c(d-Cys-Tyr-d-4-amino-Phe(carbamoyl)-Lys-Thr-Cys)-d-Tyr-NH_2_) (Fig. 15) conjugated to two macrocyclic chelators, CB-TE2A (Fig. 8) and NODAGA (Fig. 13a), and a ^64^Cu radionuclide.

Their study determined the relative influence of chelating agents and radionuclides in in vitro and in vivo studies. The results showed that, similar to agonists, changes within these two factors can have a large influence on the binding affinities and pharmacokinetics of antagonists. The internalisation levels of ^64^Cu-CB-TE2A-LM3 and ^64^Cu-NODAGA-LM3 in tumour cells presenting sstr2 were significant but still lower than those of agonists. ^64^Cu-NODAGA-LM3 is relatively hydrophilic and carries no charge. It therefore exhibited faster clearance and lower kidney uptake than ^64^Cu-CB-TE2A-LM3. The tumour-to-normal tissue ratio was also much higher with ^64^Cu-NODAGA-LM3. Both complexes, however, exhibited favourable pharmacokinetics and high image contrast [65].

## Conclusions

In presented review we demonstrated that SST agonists and antagonists in connection with radio-copper complexes, are promising tools for PRRT. Both investigated groups of SST analogues have yielded results confirming theirs suitability for clinical applications. We described herein results obtained for fourteen agonists and two antagonists of SST receptors. Some of them may be considered as alternatives to OctreoScan, which is called as the gold standard for imaging and therapy of patients with neuroendocrine tumours. However, there remains a great deal of unexplored opportunities, because until recently radiopharmaceuticals containing copper radioisotopes were not accepted for regular use in medicine.

## References

[1] Brazeau P, Vale W, Burgus R, Ling N, Butcher M, Rivier J, Guillemin R (1973). Hypothalamic polypeptide that inhibits secretion of immunoreactive pituitary growth hormone. Science.

[2] Patel YC (1999). Somatostatin and its receptor family. Front Neuroendocrinol.

[3] Barbieri F, Bajetto A, Pattarozzi A, Gatti M, Wurth R, Thellung S, Corsaro A, Villa V, Nizzari M, Florio T (2013). Peptide receptor targeting in cancer: the somatostatin paradigm. Int J Pept.

[4] Pawlikowski M (2007). Somatostatin analogs in diagnostics and therapy.

[5] Dasgupta P (2004). Somatostatin analogues: multiple roles in cellular proliferation, neoplasia, and angiogenesis. Pharmacol Ther.

[6] Watt HL, Kharmate G, Kumar U (2008). Biology of somatostatin in breast cancer. Mol Cell Endocrinol.

[7] Lamberts SWJ, Krenning EP, Reubi JC (1991). The role of somatostatin and its analogs in the diagnosis and treatment of tumors. Endocr Rev.

[8] Jacobs S, Schulz S (2008). Intracellular trafficking of somatostatin receptors. Mol Cell Endocrinol.

[9] Cholewiński W, Tarkowska A (2002). Ligandy receptorów somatostatynowychw diagnostyce i terapii radioizotopowej w onkologii. Nowotw J Oncol.

[10] Lamberts SWJ, de Herder WW, Hofland LJ (2002). Somatostatin analogs in the diagnosis and treatment of cancer. Trends Endocrinol Metab.

[11] Appetecchia M, Baldelli R (2010). Somatostatin analogues in the treatment of gastroenteropancreatic neuroendocrine tumours, current aspects and new perspectives. J Exp Clin Cancer Res.

[12] Strosberg J, Kvols L (2010). Antiproliferative effect of somatostatin analogs in gastroenteropancreatic neuroendocrine tumors. World J Gastroenterol.

[13] Okarvi SM (2008). Peptide-based radiopharmaceuticals and cytotoxic conjugates: potential tools against cancer. Cancer Treat Rev.

[14] Okarvi SM (2004). Peptide-based radiopharmaceuticals: future tools for diagnostic imaging of cancers and other diseases. Med Res Rev.

[15] Breeman WAP, de Jong M, Kwekkeboom DJ, Valkema R, Bakker WH, Kooij PPM, Visser TJ, Krenning EP (2001). Somatostatin receptor-mediated imaging and therapy: basic science, current knowledge, limitations and future perspectives. Eur J Nucl Med.

[16] Pawlikowski M, Melen-Mucha G (2003). Perspectives of new potential therapeutic applications of somatostatin analogs. Neuroendocrinol Lett.

[17] Majumdar S, Siabaan TJ (2012). Peptide-mediated targeted drug delivery. Med Res Rev.

[18] Naqvi SAR, Sosabowski JK, Nagra SA, Ishfaq MM, Mather SJ, Matzow T (2011). Radiopeptide internalisation and externalisation assays: cell viability and radioligand integrity. Appl Radiat Isot.

[19] Teunissen JJM, Kwekkeboom DJ, de Jong M, Esser JP, Valkema R, Krenning EP (2005). Peptide receptor radionuclide therapy. Best Pract Res Clin Gastroenterol.

[20] Reubi JC, Schar JC, Waser B, Wenger S, Heppeler A, Schmitt JS, Macke HR (2000). Affinity profiles for human somatostatin receptor subtypes SST1-SST5 of somatostatin radiotracers selected for scintigraphic and radiotherapeutic use. Eur J Nucl Med Mol Imaging.

[21] Bakker WH, Albert R, Bruns C, Breeman WAP, Hofland LJ, Marbach P, Pless J, Pralet D, Stolz B, Koper JW, Lamberts SWJ, Visser TJ, Krenning EP (1991). [^111^In-DTPA-D-Phe^1^]-octreotide, a potential radiopharmaceutical for imaging of somatostatin receptor-positive tumors: synthesis, radiolabeling and in vitro validation. Life Sci.

[22] Rufini V, Calcagni ML, Baum RP (2006). Imaging of neuroendocrine tumors. Semin Nucl Med.

[23] Forrer F, Valkema R, Kwekkeboom DJ, de Jong M, Krenning EP (2007). Peptide receptor radionuclide therapy. Best Pract Res Clin Endocrinol Metab.

[24] de Jong M, Bakker WH, Krenning EP, Breeman WA, van der Pluijm ME, Bernard BF, Visser TJ, Jermann E, Behe M, Powell P, Macke HR (1997). Yttrium-90 and indium-111 labelling, receptor binding and biodistribution of [DOTA0, D-Phe1, Tyr3]octreotide, a promising somatostatin analogue for radionuclide therapy. Eur J Nucl Med.

[25] Krenning EP, de Jong M, Kooij PP, Breeman WA, Bakker WH, de Herder WW, van Eijck CH, Kwekkeboom DJ, Jamar F, Pauwels S, Valkema R (1999). Radiolabelled somatostatin analogue(s) for peptide receptor scintigraphy and radionuclide therapy. Ann Oncol.

[26] Kwekkeboom DJ, Teunissen JJ, Bakker WH, Kooij PP, de Herder WW, Feelders R, van Eijck CH, Esser JP, Kam BL, Krenning EP (2005). Radiolabeled somatostatin analog [177Lu-DOTA0, Tyr3]octreotate in patients with endocrine gastroenteropancreatic tumors. J Clin Oncol.

[27] Asabella AN, Cascini GL, Altini C, Paparella D, Notaristefano A, Rubini G (2014). The copper radioisotopes: a systematic review with special interest to ^64^Cu. BioMed Res Int.

[28] Szymański P, Frączek T, Markowicz M, Mikiciuk-Olasik E (2012). Development of copper based drugs, radiopharmaceuticals and medical materials. Biometals.

[29] Smith SV (2004). Molecular imaging with copper-64. J Inorg Biochem.

[30] Wadas TJ, Wong EH, Weisman GR, Anderson CJ (2007). Copper chelation chemistry and its role in copper radiopharmaceuticals. Curr Pharm Des.

[31] Williams HA, Robinson S, Julyan P, Zweit J, Hastings D (2005). A comparison of PET imaging characteristics of various copper radioisotopes. Eur J Nucl Med Mol Imaging.

[32] Rowshanfarzad P, Sabet M, Jalilian AR, Kamalidehghan M (2006). An overview of copper radionuclides and production of ^61^Cu by proton irradiation of ^nat^Zn at a medical cyclotron. Appl Radiat Isot.

[33] Szelecsenyi F, Suzuki K, Kovacs Z, Takei M, Okada K (2002). Production possibility of ^60,61,62^Cu radioisotopes by alpha induced reactions on cobalt for PET studies. Nucl Instrum Methods B.

[34] Blower PJ, Lewis JS, Zweit J (1996). Copper radionuclides and radiopharmaceuticals in nuclear medicine. Nucl Med Biol.

[35] Jamriska DJ, Taylor WA, Ott MA, Heaton RC, Phillips DR, Fowler MM (1995). Activation rates and chemical recovery of ^67^Cu produced with low energy proton irradiation of enriched ^70^Zn targets. J Radioanal Nucl Chem.

[36] Zinn KR, Chaudhuri TR, Cheng TP, Morris JS, Meyer WA (1994). Production of no-carrier-added Cu-64 from zinc metal irradiated under born shielding. Cancer.

[37] McCarthy DW, Shefer RE, Klinkowstein RE, Bass LA, Margeneau WH, Cutler CS, Anderson CJ, Welch MJ (1997). The efficient production of high specific activity Cu-64 using a biomedical cyclotron. Nucl Med Biol.

[38] Liu S (2008). Bifunctional coupling agents for radiolabeling of biomolecules and target-specific delivery of metallic radionuclides. Adv Drug Deliv Rev.

[39] Anderson CJ, Pajeau TS, Edwards WB, Sherman ELC, Rogers BE, Welch MJ (1995). In vitro and in vivo evaluation of copper-64-octreotide conjugates. J Nucl Med.

[40] Bass LA, Wang M, Welch MJ, Anderson CJ (2000). In vivo transchelation of copper-64 from TETA-octreotide to superoxide dismutase in rat liver. Bioconjugate Chem.

[41] Anderson CJ, Jones LA, Bass LA, Sherman ELC, McCarthy DW, Cutler PD, Lanahan MV, Cristel ME, Lewis JS, Schwarz SW (1998). Radiotherapy, toxicity and dosimetry of copper-64-TETA-octreotide in tumor-bearing rats. J Nucl Med.

[42] Anderson CJ, Dehdashti F, Cutler PD, Schwarz SW, Laforest R, Bass LA, Lewis JS, McCarthy DW (2001). ^64^Cu-TETA-octreotide as a PET imaging agent for patients with neuroendocrine tumors. J Nucl Med.

[43] Dehdashti F, Anderson CJ, Trask DD, Bass LA, Schwarz SW, Cutler PD, McCarthy DW, Lanahan MV (1997). Initial results with PET imaging using Cu-64-labeled TETA-octreotide in patients with carcinoid tumor. J Nucl Med.

[44] Lewis JS, Lewis MR, Srinivasan A, Schmidt MA, Wang J, Anderson CJ (1999). Comparison of four ^64^Cu-labeled somatostatin analogues in vitro and in a tumor-bearing rat model: evaluation of new derivatives for positron emission tomography imaging and targeted radiotherapy. J Med Chem.

[45] de Jong M, Breeman WAP, Bakker WH, Kooij PPM, Bernard BF, Hofland LJ, Visser TJ, Srinivasan A, Schmidt MA, Erion JL, Bugaj JE, Macke HR, Krenning EP (1998). Comparison of ^111^In-labeled somatostatin analogues for tumor scintigraphy and radionuclide therapy. Cancer Res.

[46] De Jong M, Bakker WH, Breeman WAP, Bernard WH, Hofland LJ, Visser TJ, Srinivasan A, Schmidt M, Behe M, Macke HR, Krenning EP (1998). Preclinical comparison of [DTPA0]octreotide, [DTPA0, Tyr3]-octreotide and [DOTA, Tyr3]-octreotide as carriers for somatostatin receptor-targeted scintigraphy and radionuclide therapy. Int J Cancer.

[47] Erion JL, Srinivasan A, Schmidt MA, Wilhelm R, Bugaj JE (1997). Radiolabeled ligand-octreotate conjugates: evaluation of potential diagnostic and therapeutic radiopharmaceutical agents targeted to somatostatin receptors. J Nucl Med.

[48] Lewis JS, Srinivasan A, Schmidt MA, Anderson CJ (1999). In vitro and in vivo evaluation of ^64^Cu-TETA-Tyr^3^-octreotate. A new somatostatin analog with improved target tissue uptake. Nucl Med Biol.

[49] Lewis JS, Lewis MR, Cutler PD, Srinivasan A, Schmidt MA, Schwarz SW, Morris MM, Miller JP, Anderson CJ (1999). Radiotherapy and dosimetry of ^64^Cu-TETA-Tyr^3^-octreotate in a somatostatin positive tumor-bearing rat model. Clin Cancer Res.

[50] Sprague JE, Peng Y, Sun X, Weisman GR, Wong EH, Achilefu S, Anderson CJ (2004). Preparation and biological evaluation of copper-64-labeled Tyr^3^-octreotate using a cross-bridged macrocyclic chelator. Clin Cancer Res.

[51] Nguyen K, Parry JJ, Rogers BE, Anderson CJ (2012). Evaluation of copper-64-labeled somatostatin agonists and antagonist in SSTr2-tranfected cell lines that are positive and negative for p53: implications for cancer therapy. Nucl Med Biol.

[52] Sun X, Wuest M, Kovacs Z, Sherry AD, Motekaitis R, Wang Z, Martell AE, Welch MJ, Anderson CJ (2003). In vivo behavior of copper-64-labeled methanephosphonate tetraaza macrocyclic ligands. J Biol Inorg Chem.

[53] Guo Y, Ferdani R, Anderson CJ (2012). Preparation and Biological evaluation of ^64^Cu labeled Tyr^3^-octreotate using a phosphonic acid-based cross-bridged macrocyclic chelator. Bioconjugate Chem.

[54] Pfeifer A, Knigge U, Mortensen J, Oturai P, Berthelsen AK, Loft A, Binderup T, Rasmussen P, Elema D, Klausen TL, Holm S, von Benzon E, Hojgaard L, Kjaer A (2011). Clinical PET of neuroendocrine tumors using 64Cu-DOTATATE: first-in-humans study. J Nucl Med.

[55] Pfeifer A, Johnbeck CB, Knigge U, Mortensen J, Oturai P, Loft A, Berthelsen A, Binderup T, Rasmussen P, Kjaer A (2013). Clinical PET imaging of neuroendocrine tumors using ^64^Cu-DOTA-Tyr^3^-octreotate. J Nucl Med.

[56] Pfeifer A, Knigge U, Binderup T, Mortensen J, Oturai P, Loft A, Berthelsen AK, Langer SW, Rasmussen P, Elema D, von Benzon E, Hojgaard L, Kjaer A (2015). ^64^Cu-DOTATATE PET for neuroendocrine tumors: a prospective head-to-head comparison with ^111^In-DTPA-octreotide in 112 paients. J Nucl Med.

[57] Johnbeck CB, Knigge U, Loft A, Berthelsen AK, Mortensen J, Oturai P, Langer S, Elema D, Kjaer A (2016). Head-to-head comparison of ^64^Cu-DOTATATE and ^68^Ga-DOTATOC PET/CT: a prospective study of 59 patients with neuroendocrine tumors. J Nucl Med.

[58] Peterson BM, Roselt P, Denoyer D, Cullinane C, Binns D, Noonan W, Jeffery CM, Price RI, White JM, Hicks RJ, Donnelly PS (2014). PET imaging of tumours with a ^64^Cu labeled macrobicyclic cage amine ligand tethered to Tyr^3^-octreotate. Dalton Trans.

[59] Ullrich M, Bergmann R, Peitzsch M, Zenker EF, Cartellieri M, Bachmann M, Ehrhart-Bornstein M, Block NL, Schally AV, Eisenhofer G, Bornstein SR, Pietzsch J, Ziegler CG (2016). Multimodal somatostatin receptor theranostics using [^64^Cu]Cu-/[^177^Lu]Lu-DOTA-(Tyr^3^)octreotate and AN-238 in a mouse pheochromocytoma model. Theranostics..

[60] Cai Z, Ouyang Q, Nguyen KN, Modi J, Wang L, White AG, Rogers BE, Xie X, Anderson CJ (2014). ^64^Cu-labeled analogues conjugated with cross-bridged phosphonate-based chelators via strain-promoted click chemistry for PET imaging: in silico through in vivo studies. J Med Chem.

[61] Nedrow JR, White AG, Modi J, Nguyen K, Chang AJ, Anderson CJ (2014). Positron emission tomographic imaging of copper 64- and gallium 68-labeled chelator conjugates of the somatostatin agonist Tyr^3^-octreotate. Mol Imaging.

[62] Ginj M, Zhang H, Waser B, Cescato R, Wild D, Wang X, Erchegyi Rivier J, Macke HR, Reubi JC (2006). Radiolabeled somatostatin receptor antagonists are preferable to agonists for in vivo peptide receptor targeting of tumors. Proc Natl Acad Sci USA.

[63] Wild D, Fani M, Behe M, Brink I, Rivier JEF, Reubi JC, Maecke HR, Weber WA (2011). First clinical evidence that imaging with somatostatin receptor antagonists is feasible. J Nucl Med.

[64] Wadas TJ, Eiblmaier M, Zheleznyak A, Sherman CD, Ferdani R, Liang K, Achilefu S, Anderson CJ (2008). Preparation and biological evaluation of ^64^Cu-CB-TE2A-sst2-ANT, a somatostatin antagonist for PET imaging of somatostatin receptor-positive tumors. J Nucl Med.

[65] Fani M, Del Pozzo L, Abiraj K, Mansi R, Tamma ML, Cescato R, Waser B, Weber WA, Reubi JC, Maecke HR (2011). PET of somatostatin receptor-positive tumors using ^64^Cu- and ^68^Ga-somatostatin antagonists: the chelate makes the difference. J Nucl Med.

